# Overexpression of a Wheat Aquaporin Gene, *Td*PIP2;1, Enhances Salt and Drought Tolerance in Transgenic Durum Wheat cv. Maali

**DOI:** 10.3390/ijms20102389

**Published:** 2019-05-14

**Authors:** Malika Ayadi, Faiçal Brini, Khaled Masmoudi

**Affiliations:** Biotechnology and Plant Improvement Laboratory, Centre of Biotechnology of Sfax (CBS), University of Sfax, B.P 1177, 3018 Sfax, Tunisia; malika.ayadi@cbs.rnrt.tn (M.A.); faical.brini@cbs.rnrt.tn (F.B.)

**Keywords:** abiotic stress, antioxidant enzymes, aquaporin, *Td*PIP2;1, histochemical analysis, transgenic wheat, transpiration

## Abstract

In this study, we generated transgenic durum wheat cv. Maali overexpressing the wheat plasma membrane aquaporin *Td*PIP2;1 gene under the control of Pr*Td*PIP2;1 promoter or under the constitutive PrCaMV35S promoter. Histochemical analysis of the fusion Pr*Td*PIP2;1::*Td*PIP2;1::GusA in wheat plants showed that the β-glucuronidase (GUS) activity was detected in the leaves, stems and roots of stably transformed wheat T3 plants. Our results showed that transgenic wheat lines overexpressing the *Td*PIP2;1 gene exhibited improved germination rates and biomass production and retained low Na^+^ and high K^+^ concentrations in their shoots under high salt and osmotic stress conditions. In a long-term study under greenhouse conditions on salt or drought stress, transgenic *Td*PIP2;1 lines produced filled grains, whereas wild-type (WT) plants either died at the vegetative stage under salt stress or showed drastically reduced grain filling under drought stress. Performing real time RT-PCR experiments on wheat plants transformed with the fusion Pr*Td*PIP2;1::GusA, we showed an increase in the accumulation of GusA transcripts in the roots of plants challenged with salt and drought stress. Study of the antioxidant defence system in transgenic wheat *Td*PIP2;1 lines showed that these lines induced the antioxidative enzymes Catalase (CAT) and Superoxide dismutase (SOD) activities more efficiently than the WT plants, which is associated with lower malondialdehyde and hydrogen peroxide contents. Taken together, these results indicate the high potential of the *Td*PIP2;1 gene for reducing water evaporation from leaves (water loss) in response to water deficit through the lowering of transpiration per unit leaf area (stomatal conductance) and engineering effective drought and salt tolerance in transgenic *Td*PIP2;1 lines.

## 1. Introduction

Wheat constitutes the most widely grown and consumed cereal in the world. There is a growing imbalance between its supply and demand. The loss of fertile lands for cereal production due to climate change and water scarcity is considered major obstacles against further increases in yield. Drought stress induces different types of responses in plants [1]. It promotes oxidative damage in chloroplasts [2,3], decreases photosynthesis and metabolic reactions [4,5,6,7], induces osmotically active compound (i.e., soluble sugars and glycine betaine) and signal molecules [8,9,10] and changes in cellular lipid composition [11]. To tolerate drought stress, plants have generated different strategies such as the formation of larger and deeper root systems [12], adjustment of stomatal closure by controlling turgor pressure changes in guard cells to reduce water loss [13], accumulation of organic metabolites of low molecular weight known as compatible solutes and protective proteins [14] and enhancement of antioxidative systems [15].

Water transport across the plasma membrane is very important for the plant cell. Many reports on the relationship between plant aquaporins (AQPs) and plant water have been published [16]. Earlier research has shown that AQPs play a major role in the transportation through the membrane of different physiologically important molecules such as water, glycerol, CO_2_ and H_2_O_2_, as well as assisting with various physiological processes such as drought, salt and chilling responses [17,18,19,20]. This suggests that AQPs play an important role in transporting a large quantity of water with the minimum of energy expenditure and seem to regulate the trans-cellular transport of water [17].

AQPs are proteins belonging to the conserved family of major intrinsic proteins (MIPs) [16,21]. There are five subfamilies of plant MIPs and among them the Plasma Membrane Intrinsic Proteins, (PIPs) are the most represented. PIP proteins can be divided into two groups, PIP1 and PIP2. These proteins, when expressed in *Xenopus laevis* oocytes, exhibit different water channel activities and can interact physically, leading to an increase of the osmotic water permeability coefficient (*Pf*) of the oocyte membrane [22,23,24]. Previous reports on AQPs have demonstrated their contribution to the water permeability of root cortex cells and their involvement in osmotic water transport in the entire root system [25]. AQPs are key players in the transport of sap-assimilated elements into phloem, stomatal closure, control of cellular homeostasis and leaf movement. [21,24,26,27]. During transpiration, AQPs control the transport of water from the roots to the leaves [21,24,26,27]. Transgenic plants overexpressing AQP proteins provide promising strategies to explore the hydraulic conductance in roots and leaves, plant transpiration, stomatal aperture and gas exchange under water deficit.

Plant AQPs play an important role in water relations. Their activities regulation and gene expression, which depend on complex processes at the transcriptional, post-transcriptional, translational and post-translational levels, are considered part of the adaptation mechanisms to environmental constraints. Over the last decade, the physiological contributions of AQPs have been investigated in planta by reverse genetics. Assuming that overexpression or down-regulation of a gene can help identify its function, this kind of approach is of key importance in the field. Such studies have already provided results demonstrating the central role of AQPs in plant physiology [28]. Under abiotic stresses or other physiological or developmental changes, differential expression of AQP transcripts or proteins was observed and reported. In rice, under osmotic stress the *Os*PIP2 transcripts in roots increased substantially [29]. In contrast, under salinity stress, expression of *Os*PIP1;1 in rice increased in leaves but was reduced in roots [30]. In barley and under drought stress, the expression of *Hv*PIP2;1 was reduced in roots and enhanced in shoots [31]. Numerous studies have confirmed that modulating AQP expression in transgenic plants can increase resistance to stresses. Indeed, expression of AQP PIP1 of *Vicia faba* (*Vf*PIP1) in transgenic *Arabidopsis thaliana* enhanced drought resistance by the reduction of transpiration rates through stomatal closure [32]. Recently, it was shown that overexpression of a barley aquaporin gene, *Hv*PIP2;5, confers salt and osmotic stress tolerance in yeast and plants [33]. Moreover, transgenic banana plants overexpressing a native plasma membrane aquaporin *Musa*PIP1;2 present high levels of tolerance to different abiotic stresses [34]. In addition, it was reported that overexpression of *Mf*PIP2-7 from *Medicago falcata* promotes cold tolerance and growth under NO^−3^ deficiency in transgenic tobacco plants [35]. Conversely, the heterologous expression of *Ta*TIP2;2 in *Arabidopsis thaliana*, compromised its drought and salinity tolerance, suggesting that *Ta*TIP2;2 may be a negative regulator to abiotic stress. This was correlated with all down-regulated stress tolerance related genes acting in an ABA-independent manner, such as SOS1, SOS2, SOS3, CBF3 and DREB2A [36]. Moreover, it was reported that plasma membrane aquaporin overexpressed in transgenic tobacco increases plant vigour under favourable growth conditions but not under drought or salt stress [37]. The regulation of AQP activity and gene expression by various developmental and environmental factors, such as salinity and drought stress, relies on complex processes and signalling pathways [38].

In a previous work, we have isolated a durum wheat PIP2 gene, named *Td*PIP2;1 and have characterized its expression in *Xenopus* oocytes [39]. The generated transgenic tobacco plants overexpressing the *Td*PIP2;1 gene showed a phenotype of tolerance towards drought and salt stress [39]. To understand this tolerance mechanism at the transcriptional level, we isolated and characterized the promoter region of the *Td*PIP2;1 gene. When challenged with drought stress, the transgenic rice plants overexpressing Pr*Td*PIP2;1 in fusion with the *Td*PIP2;1 gene, showed enhanced drought stress tolerance, while WT plants were more sensitive and exhibited symptoms of wilting and chlorosis. These results suggest that expression of the *Td*PIP2;1 gene regulated by its own promoter achieves enhanced drought tolerance in transgenic rice plants [40].

In this study, we ectopically overexpressed the *Td*PIP2;1 gene under its own promoter and demonstrated that the promoter region contained all the regulatory elements required to mediate expression in transgenic durum wheat plants cv. Maali, resulting in improved tolerance to salt and drought stresses. We confirmed the role of the *Td*PIP2;1 gene in developing tolerant-crops for specific abiotic stress without any penalty phenotype.

## 2. Results

### 2.1. Production of Transgenic Wheat TdPIP2;1 Plants

Transgenic wheat plants expressing the *Td*PIP2;1 gene under a strong constitutive promoter (SP construct) or the native core promoter Pr*Td*PIP2;1 (PR construct), were generated. Eight transgenic lines for each different construct were checked and approved by PCR for their transgenic status. For each construct, three transgenic T3 homozygous lines were selected for the further evaluation of GusA transcript accumulation and the analysis of phenotypic and physiological parameters for drought and salinity stress responses in durum wheat.

### 2.2. Analysis of GUS Activity in Relation to Stress Treatment

**Figure 1:**
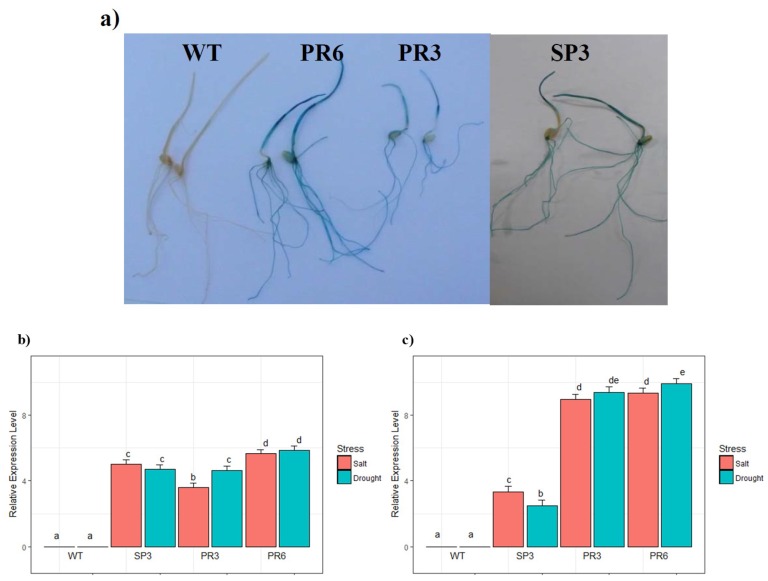
Histochemical analysis and GusA expression level of wheat transgenic lines overexpressing the *Td*PIP2;1 gene. (**a**) Histochemical GUS staining of 7 day-old wildtype (WT) and transgenic SP3, PR3 and PR6 wheat seedlings grown on MS medium. (**b**,**c**) qRT-PCR analysis of GusA gene expression in leaves and roots, respectively, of 15 day-old plantlets of WT, SP3, PR3 and PR6 lines subjected to salt (150 mM NaCl) and drought (20% PEG 6000) treatments over 48 h. Expression level of the GusA gene in control conditions was used as a reference. Bars show standard deviations of the replicates. Values represent the means of 5 replicates (*n* = 5 ± SD). Treatments with different letters have significant differences.

The histochemical staining of GUS activity for whole plants at an early developmental stage (7 day-old seedlings), grown under control conditions, uncovered a coloured product in the leaves and in roots (Figure 1a). To validate the status of stress-inducible expression of the Pr*Td*PIP2;1, GusA transcript accumulation was examined by quantitative real-time qPCR in WT, SP3, PR3 and PR6 lines grown under control conditions or subjected to salt stress treatment (150 mM NaCl) and osmotic stress (20% PEG). The changes in GusA gene expression levels, across multiple samples and stress, were carried out by comparative quantification based on the housekeeping Actin gene as a reference gene. After 48 h of stress treatment, the level of GusA transcripts in the roots was higher in transgenic lines expressing the GusA gene under the native promoter of the *Td*PIP2;1 gene (PR6 and PR3), compared to the transgenic SP3 line expressing the GUS gene under the 35S constitutive promoter (Figure 1c). In leaves, the expression level of GusA transcripts was higher in the transgenic PR6 line compared to the two other transgenic PR3 and SP3 lines (Figure 1b). For each of the two responses (relative root expression and relative leaf expression), a Two-way analysis of variance with factors stress type and genotype was carried out. The results show that the genotype effect was highly significant (*p*-value ˂ 0.001). There was a significant interaction between genotype and stress factors. The Tukey multiple comparisons procedure shows that there was a significant difference between the three genotypes, with the highest expression in roots for PR6, followed by PR3 and then SP3. For salt stress, there was no significant difference in gene expression in roots between the PR6 and the PR3 lines but both of them differed from the SP3 line. Similar results were obtained for drought stress. However, there was a significant difference for PR6 between salt and drought stress (Figure 1c). All these results suggest that the Pr*Td*PIP2;1 promoter is an abiotic and a tissue stress inducible promoter.

### 2.3. Evaluation of the Wheat TdPIP2;1 Gene for in Vitro Stress Tolerance

#### 2.3.1. Effect of Salt Stress on Seed Germination

**Figure 2:**
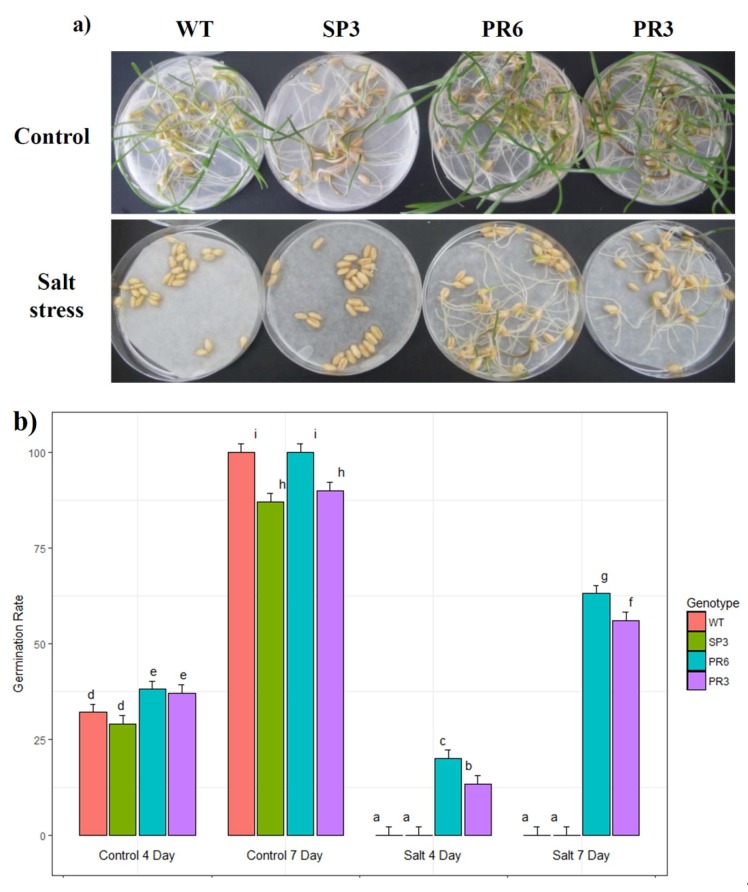
Effect of salt (NaCl 150 mM) on seed germination of transgenic wheat plants overexpressing *Td*PIP2;1 gene. (**a**) Photographs taken one week after seed germination. (**b**) Percentage of seed germination in the presence of 0 and 150 mM NaCl at 4 and 7 days of WT and T3 homozygous transgenic plants (SP3, PR3 and PR6). Values are means of 3 replicates ± SD (*n* = 3) (20 seeds per repetition). Treatments with different letters have significant differences.

In a first trial, the homozygous T3 seedlings were tested in vitro for their tolerance to salt and osmotic (mannitol) stresses. Wheat seeds from WT and three homozygous transgenic lines, SP3, PR6 and PR3, were germinated in petri-dishes with two sheets of pre-wetted filter paper supplemented with 0 or 150 mM NaCl. Under a control condition, the germination rate increased from 4 to 7 days of culture for all tested lines (Figure 2), indicating that aquaporin gene expression has no adverse effect on seed germination. However, germination of WT and SP3 seeds was significantly reduced under stress conditions when compared to transgenic PR3 and PR6 seeds. In fact, when challenged with 150 mM NaCl, the WT and SP3 seeds did not germinate, whereas after 4 days, seeds of the PR3 and PR6 lines had a germination rate of 13.3 and 20%, respectively (Figure 2). Moreover, after 7 days of salt stress treatment, seeds of the PR3 and PR6 lines showed a germination rate of 56% and 63%, respectively (Figure 2). The Two-way analysis of variance shows that the effect of stress and genotype were highly significant. PR6 lines showed the highest germination rate, followed by PR3, then WT and SP3. From the germination experiments, we noticed that the transgenic seedlings had shorter leaves and roots when grown for 7 days under salt stress.

#### 2.3.2. Effect of Salt and Osmotic Stresses on Root and Leaf Length

**Figure 3:**
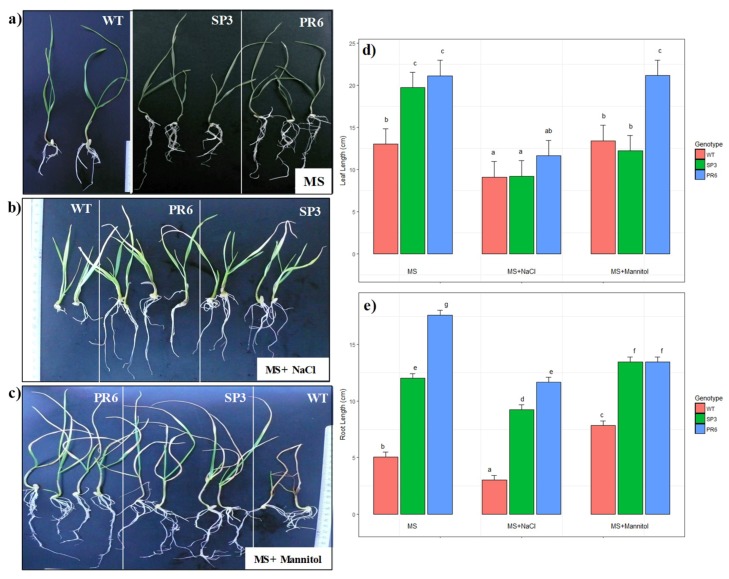
Effect of stress on growth rate of wild-type and *Td*PIP12;1 transgenic lines (SP3, PR3 and PR6). (**a**) MS medium. (**b**): MS medium supplemented with 150 mM NaCl. (**c**): MS medium supplemented with 300 mM Mannitol. (**d**,**e**) Analysis of root and leaf length of WT, SP3 and PR6 transgenic plants cultured on MS, MS + 150 mM NaCl and MS + 300 mM Mannitol, respectively. The photographs were taken 15 days after stress application. The results are expressed as the means SE of measurements from three different experiments. Values are means SD (*n* = 5). Treatments with different letters have significant differences.

In order to further explore the consequences of salt and osmotic stress on the growth rate, 3 day-old WT and transgenic (SP3 and PR6) seedlings were transferred to MS, MSs (NaCl 150 mM) or MSm (mannitol 300 mM). After an additional 10 days, WT seedlings recorded a shorter leaf and root length compared to transgenic lines in control condition (Figure 3a,d,e). Under the same condition, WT plants showed severe root length reduction of 71.3% and 58% when compared to transgenic lines PR6 and SP3, respectively, while leaf length reduction in WT plants was only 34% and 38.5% when compared to transgenic lines SP3 and PR6, respectively (Figure 3d,e). Interestingly, inhibition of root elongation under salinity stress was 40% in WT, 23% and 34% in transgenic lines SP3 and PR6, respectively. Under NaCl treatment, leaf length showed non-significant difference among the three genotypes but when compared to the control MS media, the three genotypes showed significant leaf length reduction (Figure 3d). When grown in mannitol-supplemented culture media, WT and PR6 transgenic line did not show significant leaf length reduction when compared to control MS culture media. In contrast, the SP3 transgenic line showed significant leaf length reduction in comparison with the control. Under salinity treatment, root length differed significantly among the three genotypes and when compared to the control condition, they showed significant root length reduction (Figure 3e). Seedlings conducted with mannitol-supplemented culture showed a significant increase in root length for WT and SP3 lines, while the PR6 line showed a significant decrease when compared to control condition (Figure 3e). The reduction in root length in WT was 42% when compared to transgenic lines SP3 and PR6 (Figure 3a–c). It is noteworthy to notice that reduction in root length observed in transgenic plants challenged with NaCl or mannitol stress was compensated by an augmentation in the root number when compared to control conditions (Figure 3a–c). When challenged with NaCl or mannitol stress, transgenic seedlings produced two leaves, whereas WT seedlings showed inhibition and produced only one short leaf. Leaf length was inhibited by 30% under salinity stress in WT plants when compared to control condition, while no inhibition was observed in leaf length under mannitol stress (Figure 3d). Similarly, in transgenic line PR6, leaf length inhibition under salinity stress was about 45% but no inhibition was observed under mannitol stress (Figure 3b–e). The Two-way analysis of variance shows that the effect of stress and genotype were highly significant. PR6 lines showed the highest root and leaf length under control, salt and mannitol stress conditions, followed by SP3 and then WT. The difference in leaf or root length reduction or inhibition observed between WT and transgenic wheat plants overexpressing the *Td*PIP2;1 gene under its own promoter, shows the ability of the transgenic plants to tolerate in vitro salinity and osmotic stresses.

### 2.4. Evaluation of the Wheat TdPIP2;1 Gene for Stress Tolerance under Greenhouse Conditions

In vitro growth of the transgenic wheat plants overexpressing the *Td*PIP2;1 gene showed a promising level of tolerance under salt and osmotic stresses. It was worth studying the effect of this gene on biomass and grain production. Accordingly, we carried out salt and drought stress experiments with WT and the two T3 generation of transgenic lines (PR3, PR6 and SP3) grown in soil in a greenhouse.

#### 2.4.1. Fraction of Transpirable Soil Water (FTSW)

**Figure 4:**
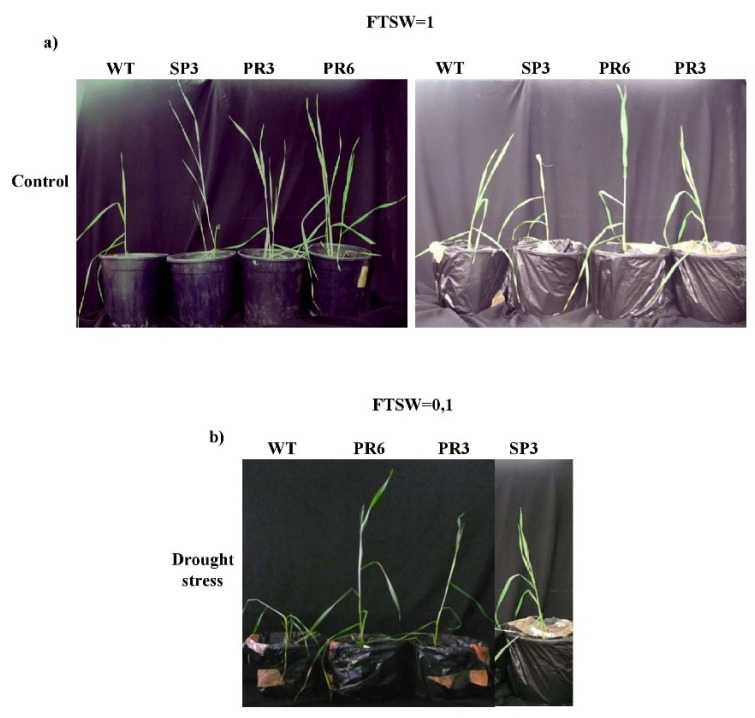
Phenotype and growth rate of WT and Pr*Td*PIP2;1 transgenic wheat lines PR6 and SP3 grown under (**a**) control conditions or subjected (**b**) to an average of 15 days of water withholding (FTSW = 0.1). Transgenic and WT wheat plants were gown in soil during 20 days in controlled greenhouse conditions. The photographs were taken 15 days after water stress application.

**Figure 5:**
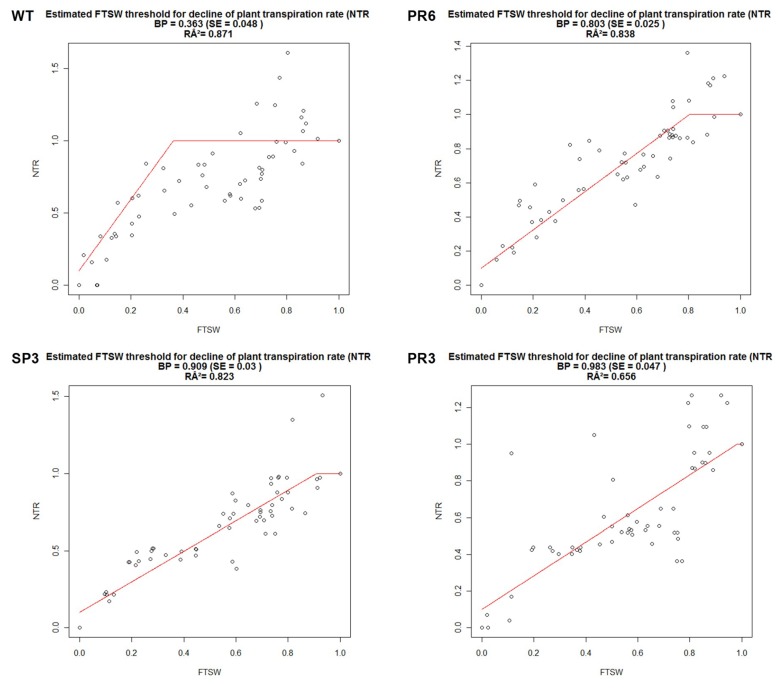
Variability for normalized transpiration rate (NTR) control under drought stress among the WT and transgenic wheat lines tested. Classification of wheat lines are from least sensitive WT, PR6 and SP3 to the most sensitive (stomata closure for a high FTSW) (PR3).

As the soil dried progressively for about 20 days, the transpiration rate (TR) was recorded for the generated transgenic wheat plants overexpressing the Pr*Td*PIP2;1 promoter in fusion with the *Td*PIP2;1 gene. For each construct of the generated transgenic T3 homozygous lines (SP3, PR3, PR6), plants were evaluated in vitro and under greenhouse conditions for their growth (Figure 4). The Normalized transpiration rate (NTR) (transpiration of stressed plants /average transpiration of control plants = Gs/Gsmax) was calculated to reflect the daily transpiration rate and FTSW was calculated to reflect the soil-water content. The relationship between NTR and FTSW correlated perfectly to the plateau regression function (Figure 5). For the NTR decline, the FTSW threshold values were close for both transgenic wheat lines, SP3 and PR6. In contrast, the FTSW thresholds for the NTR decline were higher for the transgenic PR3 lines compared to PR6 lines. The threshold for the decline of transpiration (NTR) occurred when FTSW values of about 0.363, 0.909 and 0.983 were obtained for WT, SP3 and PR3, respectively. The FTSW threshold wherein transpiration rates began to decline was calculated for each line tested using a plateau regression procedure. The determined FTSW threshold values ranged from 0.363 to 0.909 for WT and PR3 (Table 1). There was no significant trend of change of the FTSW threshold for transpiration rate decrease between SP3 and PR6 lines. However, a significant linear (r^2^ = 0.656) decline of FTSW threshold was observed with PR3. In this line, a preventive strategy to skip excessive water loss by stomata closure at high FTSW was observed. Accordingly, the PR3 line was shown to be the most tolerant to drought stress, whereas the WT line was the most sensitive to drought stress.

The same results were observed when these lines were subjected to salt stress.. When challenged with salt or drought stress, these lines exhibited a high level of tolerance. In fact, in response to water deficit, minimization of water loss constitutes a major aspect of drought tolerance and this can be reached by lowering the transpiration per unit leaf area (stomatal conductance). In this context, it is worthwhile elucidating the contribution of this protein in the tolerance mechanism to salt and drought stress by overexpressing the isolated *Td*PIP2;1 gene under its own promoter in wheat plants.

#### 2.4.2. Effect on Biomass and Grain Production

The increase in yield stability under harsh environmental and growth conditions with improved varieties adapted to salt and drought stresses would constitute a major breakthrough for farmers. To achieve this goal, we tested the generated transgenic wheat lines under greenhouse conditions for their capacity to produce normally filled grains under constant drought or salt stress. To this end, plants were grown under either optimal fresh water supply (100% Field capacity (FC)), drought stress or continuous salt stress using NaCl (150 mM) for irrigation until the end of the plant cycle.

**Figure 6:**
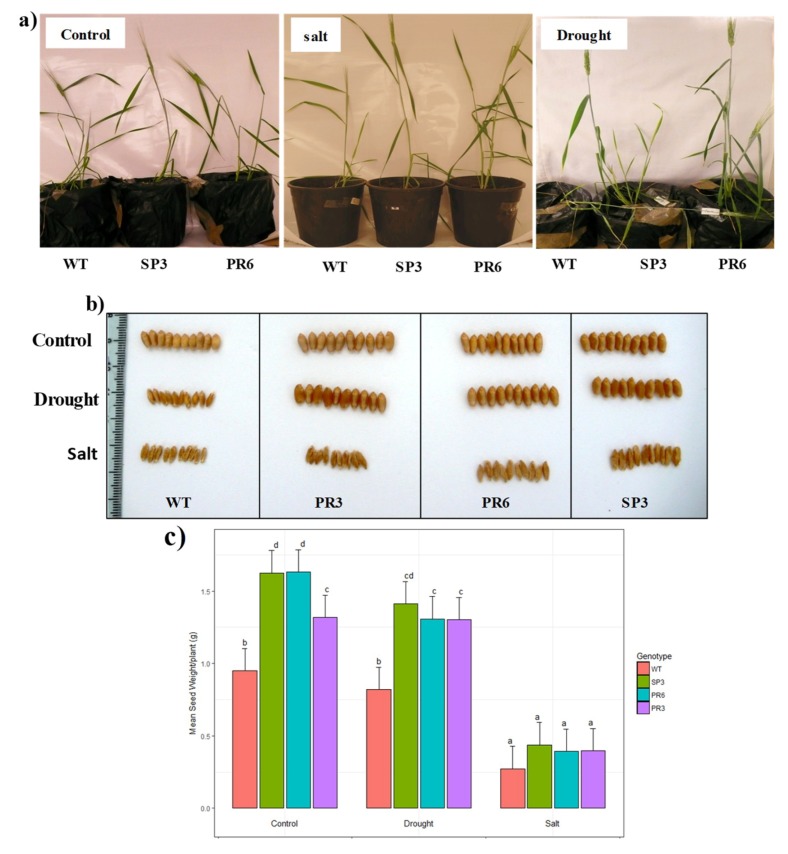
Effect of salt and drought stresses on growth and size of grains of wild-type and transgenic *Td*PIP2;1 wheat lines (SP3 and PR6). (**a**) Photographs show WT and transgenic plants grown under either normal conditions (100% FC), a continuous presence of 150 mM NaCl or a continuous drought stress (40% FC) until the end of the plant cycle. (**b**) Grain aspects of WT and transgenic (SP3, PR3 and PR6) grown under control, drought and salinity conditions. (**c**) Mean weight of seeds produced per plant under control and stress conditions. Treatments with different letters have significant differences.

The transgenic T3 generation plants of SP3, PR3 and PR6 clearly performed well and exhibited improvement of the growth parameters when compared to the WT plants under control conditions, confirming that aquaporin overexpression enhances growth and did not show any yield penalty in the transgenic plants (Figure 6).

When challenged with drought stress, the transgenic wheat plants were able to continue to grow, reached maturity, flowered and set grains; whereas the WT plants’ growth was strongly inhibited and the produced grains were poorly filled. Under salt stress, the WT plants were highly affected and showed chlorosis and a stunted phenotype, were unable to produce viable grains and ultimately died (Figure 6a,b). Under drought stress and when compared to the control condition, the decrease in the weight of 30 grains showed a non-significant difference for the WT, SP3 and PR3 lines, while the PR6 line showed a significant difference when compared to the control condition (Figure 6c). The recorded reduction in the weight of 30 grains was about 13% and 20% in transgenic lines SP3 and PR6, respectively in comparison to the obtained weight under control condition. In contrast, the PR3 line showed no reduction in the weight of 30 grains compared to the control condition. Under salinity stress, the weight of 30 grains was drastically affected in all genotypes and showed a significant difference when compared to the control condition. The reduction in the weight of 30 grains was above 70% in all genotypes compared to the control condition (Figure 6a–c).

### 2.5. Na^+^ and K^+^ Accumulation in Transgenic Wheat TdPIP2;1 Plants under Salt Stress Treatment

**Figure 7:**
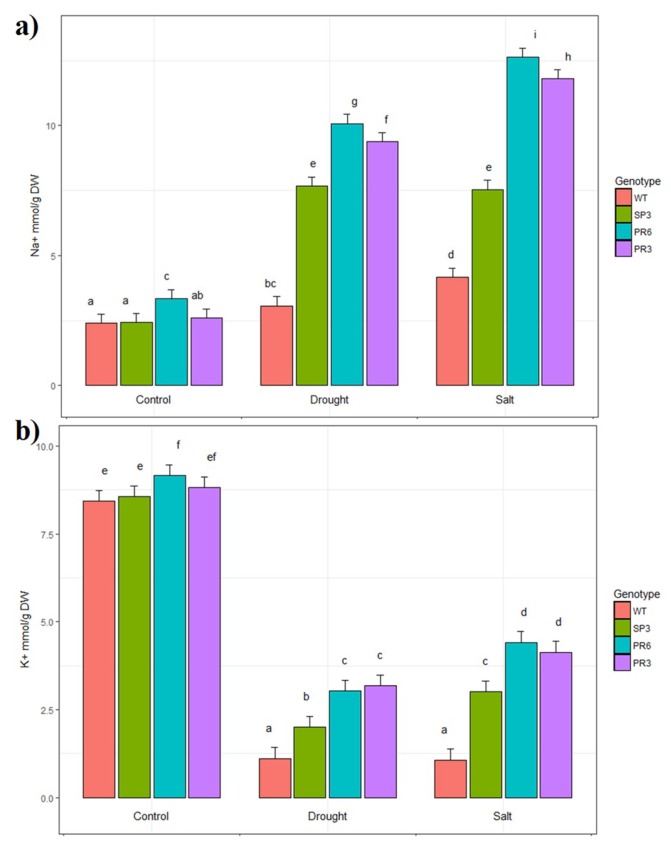
(**a**) Na^+^ and (**b**) K^+^ accumulation in leaves of the wild-type and transgenic lines overexpressing the *Td*PIP2;1 gene and grown under control or continuous presence of 150 mM NaCl or drought stress (40% FC). Values are the mean ± standard deviation (SD) (*n* = 3). Treatments with different letters have significant differences.

To understand the basic mechanism of salt and/or drought-tolerance, the endogenous Na^+^ and K^+^ contents in leaves of transgenic wheat lines overexpressing the *Td*PIP2;1 gene were monitored. Under the control condition, there was no significant difference observed in Na^+^ or K^+^ accumulation or partitioning in leaves of WT and transgenic plants, except for PR6 plants where the accumulation was slightly increased for Na^+^ (Figure 7a,b). Nevertheless, under drought or salt stress conditions, the increase of Na^+^ accumulation in leaves showed a significant difference among all genotypes when compared to control condition. Hence, the increase of Na^+^ concentration in leaves of the transgenic plants was approximately threefold that of WT (Figure 7a). Furthermore, K^+^ accumulation was higher in control condition for all genotypes compared to stress conditions and the decrease in K^+^ accumulation observed in all genotypes under drought or salt stress was highly significant compared to the control condition (Figure 7b). On the other hand, under drought or salt stress conditions, K^+^ accumulation was greater in transgenic lines than in WT (range between 1.8 to 4.13 fold) (Figure 7b). The two-way analysis of variance shows that the effect of stress and genotype were highly significant. Overall, these results suggest that transgenic plants retained selectively more K^+^ than Na^+^ in their leaves and the Na^+^/K^+^ ratio, which is an important stress tolerance trait, decreased from 3.9 in WT plants to 2.85 in PR3 and PR6 transgenic lines and to 2.5 in transgenic SP3 lines, thus preventing the young photosynthetically active organs from Na^+^ accumulation and toxicity.

### 2.6. Oxidative Stress Evaluation of Transgenic Wheat TdPIP2;1 Plants

**Figure 8:**
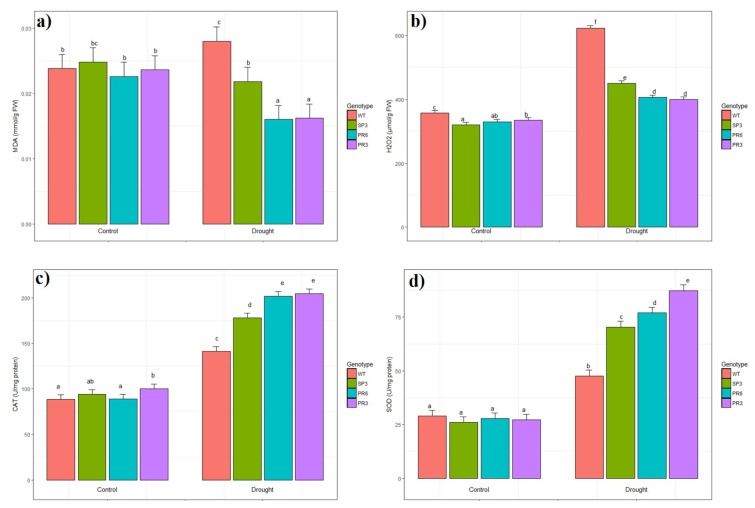
Analysis of enzymatic activities of (**a**) malondialdehyde (MDA), (**b**) hydrogen peroxide, (**c**) catalase (CAT) and (**d**) superoxide dismutase (SOD) in wild-type plants and transgenic lines (SP3, PR3 and PR6) subjected or not to drought stress. Values are means of 5 replicates ± SD (*n* = 5). Treatments with different letters have significant differences.

In the present study, Malondialdehyde (MDA) content in the leaves of WT and transgenic wheat lines correlated with drought stress induced growth inhibition. Moreover, when compared to the transgenic lines, the MDA content increased significantly in WT plants under drought stress (Figure 8a). In contrast, MDA content decreased significantly in the two transgenic lines PR6 and PR3 compared to WT plants under drought stress conditions (Figure 8a). The decrease in MDA content under drought stress was significantly different for PR3 and PR6 lines compared to control condition, while for the SP3 line the difference was not significant. Hence, transgenic wheat *Td*PIP2;1 lines with lower MDA content were shown to be more tolerant to drought.

The oxidative damage was checked by measuring the accumulation of H_2_O_2_ in leaves of the control and stressed transgenic wheat lines. As shown in Figure 8b, drought stress treatment induced significant accumulation of H_2_O_2_ content in leaves of the WT plants compared to transgenic lines (1.53 fold). Under drought stress, the increase in H_2_O_2_ content in leaves showed significant difference for all genotypes when compared to control condition (Figure 8b).

The activities of SOD and CAT, two essential antioxidant enzymes, were analysed in the leaves of WT and transgenic wheat *Td*PIP2;1 lines. When challenged with drought stress, the increase in CAT and SOD activities showed a significant difference for all genotypes compared to the control condition (Figure 8c,d). CAT and SOD activities increased significantly in transgenic lines, reaching about 2.2 and 3.2 fold, respectively relative to the non-treated plants (Figure 8c,d). However, this increase in CAT and SOD activities is lower in WT when comparing control and stress conditions (Figure 8c,d). The Two-way analysis of variance shows that the effect of stress and genotype were highly significant.

## 3. Discussion

In a large number of plant species, constitutive promoters were used to study the expression of transgenes. However, in some specific cases, constitutive expression could be damaging to the recipient plant, generating sterility, development retardation, atypical morphology, yield penalty, modified grain composition or transgene silencing [41]. To overcome the negative effects of the constitutive promoter, the use of a tissue-specific promoter is expected to be the solution for driving candidate gene expression restricted to tissues of interest and at given developmental stages. It is expected that utilization of stress inducible promoters will have a great impact on improving plant tolerance to either abiotic or biotic stress. Aquaporins (AQP) are ubiquitous membrane proteins with members exhibiting both tissue specific and inducible regulation. It has been shown that the *Nt*AQP1 gene is specifically expressed in the mesophyll tissue of *Arabidopsis thaliana* and plays an important role in increasing both net photosynthesis and mesophyll CO_2_ conductance. Moreover, the *Nt*AQP1 gene targeted at the cells of the vascular envelope significantly improved the plants’ stress response [42].

In this report, we demonstrated that the overexpression of the *Td*PIP2;1 gene in transgenic durum wheat homozygous lines significantly enhanced tolerance under constant salt and drought stresses (Figure 4). These results affirm our previous findings in rice [40]. In fact, transgenic rice plants transformed with the Pr*Td*PIP2;1 fused to the *Td*PIP2;1 gene and treated with either salt or drought stress, exhibited growth and vigour enhancement when compared to wild-type plants and therefore showed higher tolerance to abiotic stresses [40]. Our results were similar to those observed in soybean, where the overexpression of *Gm*PIP2;9 under a native promoter increased tolerance to drought stress in both solution and soil plots. This was correlated with expanded net CO_2_ assimilation of photosynthesis, stomata conductance and transpiration rate [43].

Characterization of transgenic (SP3) wheat plants transformed with the 35S promoter fused to the *Td*PIP2;1 gene and cultivated in vitro under salt and drought stress showed enhanced growth and vigour compared to wild-type plants (Figure 3b,c). In our preceding work on the *Td*PIP2;1 gene, similar results were reported where the expression of the *Td*PIP2;1 gene was positively correlated with stress response pathways in transgenic Tobacco plants [39] and rice plants [40].

Our analysis using transcriptional (Pr*Td*PIP2;1::GUS) and translational (Pr*Td*PIP2;1:: *Td*PIP2;1::GUS) fusions demonstrated that the *Td*PIP2;1 gene is expressed in the homologous transgenic wheat system. Using a histochemical staining technique, we were able to show that the Pr*Td*PIP2;1 promoter can drive GusA reporter gene expression with inducible and tissue-specific patterns (Figure 1a). The differential expression of AQP transcripts or proteins in response to abiotic stress or other physiological or developmental changes was reported in a number of studies (reviewed in Reference [44]). However, the transcriptional regulation of these genes is still elusive. The analysis of the promoter sequences of a variety of PIP and TIP genes from rice, maize and *A. thaliana* has shown the presence of putative regulatory elements such as DREs (drought responsive elements), LTREs (low temperature responsive elements) and ABREs (ABA responsive elements) [44,45]. In wheat, the *Td*PIP2;1 gene sequence revealed the presence of three highly conserved motifs in positions (261(+) CNGTTR; 435(+) CNGTTR; and 526(+) CNGTTR or CANNTG); identified at MYBCORE transcription factor binding sites responsive to water stress and induced by dehydration [36]. In addition, the sequence analysis of pro*Td*PIP2;1 revealed the presence of WRKY transcription factor required for positive and negative regulation of abscisic acid signalling [40]. This indicates the regulation of these genes at a transcriptional level in response to abiotic stresses [44,45].

To confirm the results obtained in vitro for the transgenic wheat plants transformed with the *Td*PIP2;1 gene driven by its own promoter (PR3 and PR6) or the constitutive 35S promoter (SP3) under salt and osmotic (mannitol) stress, we designed an experiment under greenhouse conditions and tested these transgenic plants for abiotic stress tolerance. Our results showed that transpiration rates were affected in control wheat plants under drought stress by closure of stomata and changes in leaf morphology. If drought stress was initiated at the vegetative phase, the first perceived response was a relatively quick decrease in leaf expansion, leaf rolling and stomata closure (Figure 4). Interestingly, the PR3 line, which is the most sensitive to stomata closure for a high FTSW, was the most tolerant line to drought stress compared to SP3 and PR6 (Figure 5). In this case, there was a significant linear decline of the FTSW threshold (r^2^ = 0.656).

Under abiotic stress, oxidative damage can be induced in many plants such as pea, rice and tomato [46,47]. ROS degrades polyunsaturated lipids forming MDA which is considered a biomarker of the level of lipid peroxidation. Accordingly, our results indicated that drought stress induced oxidative stress in transgenic *Td*PIP2;1 wheat lines manifested by low MDA content and high H_2_O_2_ contents (Figure 8a,b). Nevertheless, under drought stress, the lipid peroxidation level and H_2_O_2_ content were higher in WT than in transgenic lines SP3, PR3 and PR6. These data suggest that the transgenic wheat lines were preserved against oxidative damage under drought stress.

For the antioxidant enzymes activities, drought stress induced up-regulation of the activities in WT and transgenic wheat lines. When comparing the enzymatic activity of these genotypes, higher activities were obtained in the transgenic lines than in WT (Figure 8c,d). This suggested that transgenic lines present a higher capacity for scavenging ROS than WT under stress conditions. Consequently, the reduction in the content of H_2_O_2_ in transgenic lines is the result of the SOD reaction, which is followed by an increased enzymatic activity to catalyse the dismutation of the superoxide radical. The correlation between abiotic stress tolerance and an increase in SOD activity has been reported in many previous works [48,49,50,51,52]. Overall, it seems that the salt tolerance phenotype of transgenic wheat lines could be the result of either a low production of ROS or a better capacity to prevent ROS than the WT plants.

## 4. Materials and methods

### 4.1. Plant Material

Wheat seeds *(Triticum turgidum* L. subsp. *durum*), cv. Maali (New seed lot) were supplied by INRAT, Laboratoire de Physiologie Végétale (Tunis, Tunisia) and were used for genetic transformation.

### 4.2. Construction of the Binary Vector and Wheat Transformation Procedure

To perform gene expression and histochemical analysis, we used translational fusion with the reporter GUS gene, Pr*Td*PIP2;1::*Td*PIP2;1::GUS (named PR) and CaMV35S::*Td*PIP2;1::GUS (named SP) [40]. The resulting constructs were then transferred into *Agrobacterium tumefaciens* hyper-virulent strain EHA105 [53] using the freeze–thaw method [54] and finally used for durum wheat transformation experiments. The transformation method employed in this study was slightly modified from the applied protocol to bread wheat [55]. *A. tumefaciens* strains, harbouring the resulting constructs (PR and SP), were cultured on MG/L agar medium [56] containing Hygromycin (50 µg/mL) and Rifampicin (10 µg/mL) for two days at 28 °C. Bacteria were harvested when they reached an OD_600_ = 1 and were suspended in an R_2_ co-cultivation liquid medium (R_2_CL, pH 5.2) containing R_2_ Basic medium [57], glucose 10 g/L and 100 µM acetosyringone. An aliquot of 5 mL from this culture was diluted five times with the same R_2_CL medium containing the appropriate antibiotics and kept growing at 28 °C with shaking until OD_600_ reached 0.4–0.6. Durum wheat seeds cv. Maali were soaked first in 70% (*v*/*v*) ethanol for 3 min, then in sodium hypochlorite 1% (*v*/*v*) with gentle shaking for 20 min for sterilization. After that, seeds were washed five times with sterile distilled water. Seeds were additionally soaked at 4 °C in 0.1% (*v*/*v*) Pelt 44 (thiophanate-methyl at 450 g/L) fungicide (Bayer CropScience) and then germinated on wet filter paper with R2 basic medium at 25 °C for 2 days in the dark. A needle (Terumo Syringe 1 mL Insulin (U100) with 29 g × 0.5 needle ×100: BS-N1H2913) that had been previously filled with *A. tumefaciens* inoculum solution, was used to pierce twice, to a depth of approximately 1 mm, the embryonic apical meristem from the 2-d-old seedlings. The inoculated seeds were then co-cultivated on wet filter paper at 25 °C for an additional two days in the dark to avoid overgrowth of the Agrobacterium on the seeds. To eliminate the inoculum of Agrobacterium, seeds were dipped overnight at 4 °C in an aqueous solution containing Cefotaxime (1 g/L). Eventually, seeds were immersed in sodium hypochlorite 0.1% (*v*/*v*) for 15 min and then rinsed with sterile distilled water for five times. Ultimately, the inoculated wheat seeds were sown in pots filled with a mixture of sand (50%) and peat (50%), allowed to grow to maturity and to produce seeds (T1) under greenhouse conditions. The regenerated hygromycin-resistant wheat plants (T1) were numbered and named as indicated above (SP and PR). For the control, we used the WT wheat plants.

### 4.3. Histochemical GUS Staining

To help understand the presence of staining in tissues for GUS activity, a histochemical assay was carried out by incubating wheat seedlings under vacuum infiltration in 50 mM Na_2_HPO_4_ buffer (pH 7.0), 0.5 mM K_3_(Fe[CN]_6_), 0.5 mM K_4_(Fe[CN]_6_), 0.1% Triton X-100 and 1 mg/L X-Gluc (5-bromo-4-chloro-3-indolyl β-d-glucuronide cyclohexyl ammonium salt) for several minutes and then keeping them overnight at 37 °C. The chlorophyll and pigments were eliminated by soaking the stained seedlings tissues for several hours in 70% ethanol. Finally, the destained tissues were photographed. Transgenic plants overexpressing the *Td*PIP2;1 gene driven by the CaMV35S promoter and WT wheat plants were considered the positive and negative controls, respectively.

### 4.4. GusA Expression Analysis by the Quantitative Real-Time qPCR

Transcription levels of the *GusA* gene under the control of the Pr*Td*PIP2;1 or CaMV35S promoters were assessed by real-time qPCR in T3 homozygous transgenic wheat plants (leaves and roots of two-week-old plants) treated with 150 mM NaCl or 20% PEG 6000 for 48 h. The Trizol method (Invitrogen) was employed to extract total RNA from 100 mg plant tissues in accordance with the manufacturer’s recommendations [39]. To check the concentration and the quality of the extracted RNA, agarose gel (1% RNase-free) and spectrophotometer measurements (260/280 nm) were performed. To synthesize first strand cDNA, 3µg of total RNA was treated with DNase I (Promega, USA) and then reverse transcribed using oligo (dT)18 and SuperScript II reverse transcriptase (Invitrogen, Canada), per the supplier’s recommendations. Primer 3 software was used to identify the primer pairs that amplify fragments of GusA and the housekeeping actin genes. The primers used for real-time qPCR were: qGusAF, qGusAR, q-Act-F and q-Act-R, (Table 2). PCR reactions were performed in a 10 µL final volume containing 4 μL cDNA (obtained from 40 ng of DNase-treated RNA), 0.5 μL of each primer (at 10 μM), 5 µL 2× SYBR Green I master mix and 1μL of RNase-free water (Sigma, Canada). The reaction consisted of an initial denaturation step at 94 °C for 10 min followed by 45 cycles composed of 10 s at 94 °C, 10 s at 60 °C and 15 s at 72 °C, then a melting curve (5 s at 95 °C, 1 min at 65 °C and 5 min with temperature increasing from 65 °C to 97 °C). For each experimental condition three biological repetitions were performed, with three technical repetitions for each sample. The threshold cycle (CT) values of the triplicate PCRs were averaged and used for transcripts quantification at the end of the reaction. The relative expression ratio of the GusA gene was calculated by using the comparative CT method with the actin gene as an internal expression standard [58]. The relative expression level was calculated from triplicate measurements based on the 2-∆∆*C*T, where ∆∆*C*T = (CT, Target gene—CT, Actin) stressed—(CT, Target gene—CT, Actin) control. Relative expression ratios from three independent experiments (three biological repetitions) were reported.

### 4.5. Evaluation of Transgenic Wheat Plants for Abiotic Stress Tolerance

#### 4.5.1. In Vitro Assays

Seeds of wild type and transgenic homozygous T3 generation of durum wheat cv. Maali were employed for abiotic stress assays. For three experimental repetitions, thirty seeds from WT and transgenic plants were surface-sterilized by soaking in 70% ethanol for 1 min, washed with sterile distilled water and treated with 40% solution of sodium hypochlorite for 30 min. Finally, seeds were rinsed five times with sterile distilled water. Seeds were then incubated in sterile distilled water and maintained in a growth chamber (16 h of light per day, 500 μE m^−2^ S^−1^, 28 °C/25 °C day/night) for germination and growth rate parameters. After 2 days, seeds from T3 transgenic lines (PR and SP) and WT plant were sown in Petri dishes on two layers of filter paper saturated with distilled water, supplemented with 0 or 150 mM NaCl. Plates were incubated at 25 °C in a growth chamber under a 16 h light/8 h dark photoperiod. Germination rates were recorded after one week of incubation. The seed germination experiment was repeated three times. Young seedlings were transplanted to a new medium supplemented with 150 mM NaCl or 300 mM Mannitol to estimate the growth rate under osmotic or salt stress conditions. These dishes were removed and placed vertically in a growth chamber. After 15 days of culture, root/shoot lengths were determined for WT and transgenic seedlings using the UTHSCSA Image Tool, a free image processing and analysis program used to acquire, display, edit and analyse images (available online: http://www.ddsdx.uthscsa.edu/dig/iTdesc.html).

#### 4.5.2. Greenhouse Assays

To assess the effect of salt and drought treatments on biomass production, seeds from WT and transgenic lines (SP and PR) were germinated under greenhouse conditions. To initiate salt treatments, pots were irrigated with water supplemented with 150 mM NaCl. Soil moisture regimes were monitored daily by weighing the pots. For both salt and drought treatments, seeds were allowed to germinate and to grow for additional 20 days before harvesting the plants to measure their fresh weight, dry weight and plant transpiration rate. Seeds from T3 transgenic lines (PR and SP) and WT plants were sown in pots filled with peat moss and grown for 2 weeks before challenging them with stress treatments. Under control conditions, pots were irrigated weekly with fresh water to maintain plants at 100% FC. For salt stress treatment, the same irrigation program was used for watering the plants with addition of 150 mM NaCl. This NaCl concentration was maintained until the end of the plant cycle. Young leaves (top) and old leaves (bottom), were collected from salt-stressed and control plants and then dried at 80 °C for 24 h. Finally, the dried material was incubated in 0.5% HNO_3_ for a week. The Na^+^, Ca^2+^ and K^+^ contents were analysed in the filtrate using atomic absorption spectrophotometry. For drought stress, plants were maintained at 40% FC until the end of plant cycle. This was achieved by refiling the exact amount of water lost by evapotranspiration.

#### 4.5.3. Water Treatments

To monitor the soil water status, we used the fraction of transpirable soil water (FTSW). According to Sinclair et al. [59], when water stress is expressed as FTSW, plants respond to the progressive drying of soil in a similar manner.

Calculation of each pot’s FTSW value was carried out according to Sinclair and Ludlow’s method [60]. First, pots were fully watered daily and drained overnight until a day prior to initiating measurements. After one day of drainage, all pots were weighted to assess their initial water holding capacity. The total transpirable soil water (TTSW) was obtained by calculating the difference between initial pot capacities (Wi) and final pot weight after soil desiccation (Wf). The ratio of actual transpirable soil water (ATSW) to TTSW allowed the estimation of FTSW, where ATSW was the mass difference between daily (Wt) and final pot weight. A single drought cycle was implemented in half of the pots, when the plants had developed 8 leaves (five pots for each genotype).


FTSW = ATSW/TTSW = (Wt − Wf)/(Wi − Wf)
(1)

TTSW: total transpirable soil water; ATSW: actual transpirable soil water.

To restore the daily water loss, the remaining five control pots were fully watered in late afternoon. In contrast, for the stressed pots, water deficit was maintained by withholding irrigation and covering the pots with a plastic bag in order to prevent soil evaporation. Original FTSW values were assigned a value of 1. When a transpiration rate of less than 10% was reached for each stressed pot in comparison to the control, the experiment was terminated [60].

#### 4.5.4. Plant Transpiration Rate

Plant transpiration (TRj) per unit leaf area (mmol·m^−2^·s^−1^) was determined every day for each pot as the mass difference between weights every 24 h (including the watering for unstressed pots) and were divided by plant total leaf area on the previous day. At the same time, stomatal conductance (mmol·m^−2^·s^−1^) was measured each morning between 9:00 a.m. and 12:00 p.m. on the last panicle leaf per plant. These measures were acquired with a porometer (type AP4-UM-3) on the control and treated plants. Conductance measurements were performed on leaves and on its abaxial face, where stomatal density is greater.


Normalized transpiration rate (NTR) = transpiration of stressed plants/average transpiration of control plants = (Gs/Gsmax)
(2)

Porometry was also used to determine the last day of measurements (when gs(stressed)/gs(control) was less than 0.1), allowing us to estimate the value of the TTSW of each pot. The mean TTSW was remarkably stable.

### 4.6. Whole Plant Response Modelling to Increasing Water Deficit

To monitor the daily transpiration rate fluctuation due to evaporative demand changes, the drought treatment values were divided by the corresponding mean values on the well-watered (control) treatment in order to calculate the relative TR values daily. When the soil was still moist, daily TR ratio values for FTSW > 0.6 were adjusted to a mean value of 1, in order to minimize the effect of variation in initial plant size variation. It was essential to normalize the calculation of plant transpiration (NTR) for the accuracy of a further linear model. A two-slope linear relation with one parameter (FTSWt) was used to meet the plant responses to water deficit, indicating the FTSW threshold below which conductance starts decreasing. By applying a two-slope linear model to the experimental data, the parameter FTSWt was thus estimated (Equation (3)):

if FTSW ≥ FTSWt y = 1; else y = 1/FTSWt × FTSW; y being NTR
(3)

### 4.7. Lipid Peroxidation

By using the method of Ben Amor et al. [49] to calculate the amount of MDA in the leaves, the extent of lipid peroxidation was estimated. 1.0 mL of 0.5% (*w*/*v*) thiobarbituric acid (TBA) in 20% (*w*/*v*) TCA solution was added to an aliquot of 0.5 mL from the homogenate of fresh shoots in 0.1% (*w*/*v*) trichloroacetic acid (TCA) solution. Subsequently, the mixture was incubated at 90 °C for 30 min and then cooled on ice. By measuring absorbance at 532 and 600 nm in reference to an MDA standard curve, the equivalent MDA was calculated.

### 4.8. Quantitative H_2_O_2_ Measurement

The concentration of H_2_O_2_ was estimated according to the method described by Velikova et al. [61]. Fresh shoots tissue (0.5 g) was homogenized with 5 mL of 0.1% (*w*/*v*) TCA. This homogenate was then centrifuged at 12,000× *g* for 15 min. 10 mM phosphate buffer (pH 7.0) and 1 M potassium iodide were added to 0.5 mL of the supernatant. The mixture was then vortexed and its absorbance at 390 nm was recorded and H_2_O_2_ content was calculated using a standard curve with concentration ranging from 0.05 to 0.1 mM.

### 4.9. Enzyme Assays

Aliquots of frozen fresh shoot material (0.5 g) were ground to a fine powder with liquid nitrogen and homogenized in a cold solution containing 100 mM Tris–HCl buffer (pH 8.0), 10 mM EDTA (Ethylenediaminetetraacetic acid), 50 mM KCl, 20 mM MgCl_2_, 0.5 mM PMSF (Phenyl methyl sulfonyl fluoride) and 2% (*w*/*v*) PVP (Polyvinylpyrolidone). The homogenate was centrifuged at 14,000× *g* for 30 min at 4 °C and the supernatant was used for determination of the antioxidative enzyme activities.

Total catalase (CAT) activity was measured according to the method of Aebi [62], by monitoring the decline in absorbance at 240 nm as H_2_O_2_ was consumed. An aliquot of crude enzyme extract was added to the reaction mixtures containing 50 mM phosphate buffer (pH 7), 30 mM H_2_O_2_. One unit of CAT was defined as 1 µmol mL^−1^ H_2_O_2_ decomposed per minute.

Total superoxide dismutase (SOD) activity was determined by measuring the percentage of inhibition of the pyrogallol autoxidation [63]. A crude enzyme extract aliquot was added to the reaction mixture containing 10 mM pyrogallol in Tris-cacodylic acidediethylene triamine penta acetic acid buffer (pH 7.4–8). One unit of SOD was defined as the enzyme quantity required to inhibit 50% of the pyrogallol autoxidation.

### 4.10. Statistical Analysis

All data analysis was carried out using the Two-way Analysis of Variance (ANOVA) with SPSS package. Means were compared using Tukey’s (HSD) multiple comparison procedure.

## 5. Conclusions

In our previously published works, we reported that the expression of the *Td*PIP2;1 gene was strongly associated with abiotic stress response pathways in Tobacco [39] and rice [40]. In the present study, we found similar results, confirming that the expression of the *Td*PIP2;1 gene was closely related to abiotic stress response pathways in durum wheat, a staple crop, without causing any undesirable growth phenotypes or yield penalty. In addition, we found that mannitol, a major photosynthetic product in many higher plants, enhanced the activity of the *Td*PIP2;1 promoter, suggesting that Pr*Td*PIP2;1 may be involved in dehydration response. Our results provide insights for the *Td*PIP2;1 gene regulated by its own promoter which generates enhanced drought tolerance in transgenic wheat. In general, regulation of the activity and gene expression of aquaporins are considered part of the adaptation mechanisms to stress conditions through complex processes. Further studies are required to gain a deeper understanding of the function of this gene, particularly through transcriptome profiling and identification of *Td*PIP2;1 interacting proteins in plant PIPs. Recent advances in genome editing with CRISPR/Cas9 was efficiently used in combination with haploid induction (HI) to induce edits in nascent seeds of diverse monocot species [64]. HI enables widespread application of genome editing technology for crop improvement and offers a new path to understand the stress resistance mechanisms, when generating knockout of down-regulated AQP genes in wheat.

## Figures and Tables

**Table 1:** FTSW threshold values.

	BP	(R^2^)
WT	0.363	0.871
SP3	0.909	0.823
PR3	0.983	0.656
PR6	0.803	0.838

**Table 2:** List of primers used in real-time PCR.

Primers	Nucleotides Sequences (5′-3′)
Act F:	TGCATAGAGGGAAAGCACG
Act R:	AACCCAAAAGCCAACAACAGAGA
q-GusF:	CACGCCGTATGTTATTGCCG
q-GusR:	TCTTGCCGTTTTCGTGGGTA
